# Yaws in the Philippines: A clinico-seroprevalence study of selected communities in Mindanao

**DOI:** 10.1371/journal.pntd.0010447

**Published:** 2022-06-01

**Authors:** Belen Lardizabal Dofitas, Sherjan P. Kalim, Camille B. Toledo, Jan Hendrik Richardus

**Affiliations:** 1 Department of Dermatology, College of Medicine, University of the Philippines Manila, Metro Manila, Philippines; 2 Department of Public Health, Erasmus MC, University Medical Center Rotterdam, Rotterdam, The Netherlands; 3 Department of Pathology, Cotabato Regional and Medical Center, Cotabato City, Philippines; 4 Institute of Psychiatry and Behavioral Medicine, Southern Philippines Medical Center, Davao City, Philippines; London School of Hygiene and Tropical Medicine, UNITED KINGDOM

## Abstract

**Background:**

Yaws is a chronic, highly contagious skin and bone infection affecting children living in impoverished, remote communities. It is caused by *Treponema pallidum* subsp. *pertenue*. We report the prevalence of active yaws among elementary schoolchildren based on clinical and serological criteria in selected municipalities of Southern Philippines.

**Methods:**

From January to March 2017, exploratory cross-sectional surveys and screening of skin diseases were conducted in the Liguasan Marsh area of the provinces Maguindanao, Sultan Kudarat, and Cotabato. We included 9 municipalities and randomly selected one public elementary school per municipality. Members of students’ households and the communities were also examined and treated. Yaws suspects and contacts had blood tests for treponemal and non-treponemal antibodies using Dual Pathway Platform and *Treponema pallidum* particle agglutination (TPPA) tests.

**Results:**

A total of 2779 children and adults were screened for any skin disease: 2291 students, 393 household members, and 95 community members. Among 210 yaws suspects and contacts, 150 consented to serologic tests. The estimated prevalence of active yaws among schoolchildren screened was 1 out of 2291 (0.04%). Among 2532 children who were 14 years old and younger, 4 (0.2%) had active yaws. Eight adult household contacts and community members had latent yaws and 2 had past yaws. Five out of 9 municipalities were endemic for yaws.

**Conclusions:**

This study confirmed that the Philippines is endemic for yaws but at a low level in the schools surveyed. This is an under-estimation due to the limited sampling. The lack of proper disease surveillance after the eradication campaign in the 1960’s has made yaws a forgotten disease and has led to its resurgence. Yaws surveillance is needed to determine the extent of yaws in the Philippines and to help develop a strategy to eradicate yaws by 2030.

## Author summary

Yaws is a chronic, highly contagious skin and bone infection affecting children living in impoverished, remote communities. It is caused by bacteria called *Treponema pallidum* subspecie *pertenue*. To determine the prevalence of yaws, we conducted cross-sectional surveys and integrated screening of skin diseases and blood tests in nine selected schools and students’ households in three provinces located in Liguasan Marsh, Mindanao Island, Southern Philippines. A total of 2779 children and adults were screened for any skin disease: 2291 students, 393 household members, and 95 community members. Among 210 yaws suspects and contacts, 150 consented to serologic tests. The estimated prevalence of yaws among schoolchildren screened was 1 out of 2291 (0.04%). Among 2532 children who were 14 years old and younger, 4 (0.2%) had active yaws. Eight adult household contacts and community members had latent yaws and 2 had past yaws. Five out of 9 municipalities were endemic for yaws. This study confirmed that the Philippines is endemic for yaws. The occurrence among schoolchildren in the study sites was low probably due to limited sampling. A yaws surveillance and eradication program is needed for endemic communities in the Philippines. Yaws could be unreported in other previously endemic countries as well because the skin and bone signs are not recognized anymore.

## Introduction

Yaws is a chronic, contagious, nonvenereal, treponemal infection that manifests mainly in human skin as verrucous, raspberry-like nodules or plaques with moist, yellowish surfaces or may be ulcerated. Yaws mainly occurs in children younger than 15 years. Infection with *Treponema pertenue*, a subspecies of *Treponema pallidum*, causes the disease, which occurs primarily in warm, humid, tropical areas of Africa, Asia, South America, and Pacific Islands among poor rural populations where conditions of overcrowding, poor sanitation and inadequate water supply prevail. [1]

A systematic review by Mitja *et al*. in 2015 estimated that the prevalence of active yaws ranged from 0·31% to 14·54% in yaws-endemic areas while latent yaws ranged from 2·45% to 31·05%. In 2010–2013, 13 endemic countries reported 256,343 cases, of which 215,308 (84%) were from three countries; Papua New Guinea, Solomon Islands, and Ghana. The authors estimated that over 89 million people were living in yaws-endemic districts in 2012. [2]

In the 1950s, there were control programs established in Cambodia, Laos, Malaysia, and the Philippines, but no prevalence data is available for these previously endemic countries. [3] The World Health Organization (WHO) reports that at least 76 countries and territories that were endemic for yaws in the 1950s still need to be assessed to determine if the disease is still present. [1] The Philippines was one of these countries that had an "unknown" status of yaws since the last report in 1973.

Yaws in the Philippines was an important public health problem during the Spanish colonial period and in the early 1900s. The Bureau of Health in 1907 noted yaws among leprosy patients from various parts of the country who were transferred to the Culion Leprosarium. Yaws was historically found in most parts of the Philippines, affecting 10–30% of the population in certain provinces, with most cases noted among indigenous Filipinos in the Mindanao region located in the Southern Philippines. [4]

At the start of the yaws eradication campaign in 1951, the Philippines was moderately endemic with a prevalence of 9.6%. This fell to 0.4% by the end of 1960 at the end of the campaign and was deemed almost eradicated. [5] An electronic search in PubMed up to December 2019 did not reveal any report of yaws in the Philippines since the yaws program evaluation report published in 1965. A search in the Philippine Health Statistics reports of the Philippine Bureau of Health from 1961–1973 included yaws as a notifiable disease. The Philippine Health Statistics reported 3864 yaws cases (13.5/100,000 population) in 1961. [6] The last official report of yaws in the Philippines recorded 424 cases (1.1/100,000 population) in 1973. After 1973, the Philippine Health Statistics ceased reporting on yaws. [7]

Yaws had been reportedly common in the Bangsamoro Autonomous Region in Muslim Mindanao (BARMM) based on a skin survey conducted in 1999–2000 in the Liguasan Marsh area. A serologic survey conducted in 2012 found a clustering of most yaws cases in communities situated at the Liguasan Marsh, the largest swamp and marsh area in Mindanao. [8] Due to the sightings of yaws cases in Mindanao, the Department of Health commissioned a study to assess the presence of yaws in the country and to estimate its prevalence in provinces where yaws had reportedly been existing. The study findings were to be used to guide the development of a yaws control and eradication program in the Philippines.

In this paper, we report the prevalence of active yaws infection among schoolchildren in selected municipalities of the Liguasan Marsh area of three provinces: Maguindanao, Sultan Kudarat, and Cotabato based on medical history, clinical, and serological criteria. The first reported cases of yaws in the Philippines since the 1970s were detected during these surveys and the details have been published in a separate article. [9]

## Methods

### Ethics statement

The study proposal was registered with and approved by the Technical Review Committee of the Philippine Council for Health Research and Development (PHRR No. 1361). Ethical approval was granted by the St. Cabrini Medical Center-Asian Eye Institute Ethics Review Committee (SCMC-AEI ERC No. 2016–022). Written informed consent to participate in the study were secured from the parents or guardians of minors (18 years old and younger) and from adult participants. Written informed assent were secured from minors aged 6–18 years of age.

### Study design

A focused clinico-seroprevalence cross-sectional survey was carried out from February to May 2017 in the southern region of the Philippines in a major island group, Mindanao. Three municipalities per province situated in Liguasan Marsh were purposively chosen based on previous yaws sightings reported by Dofitas and Kalim during separate surveys. [8] We conducted school-based surveys in one randomly selected public elementary school per selected municipality, totaling 9 schools. In addition, we examined household members and community members during the skin clinics. We also conducted key informant interviews of selected respondents to find out where else yaws cases had been sighted.

### Setting

*Mindanao* is one of the three island groups making up the Philippine Archipelago. Its largest island is the Mindanao landmass. The population is over 24 million or 23.9% of the total country’s population as of 2015. [10] Liguasan Marsh is a centrally located, large catchment basin in Mindanao, found within three adjacent provinces: Maguindanao, Cotabato, and Sultan Kudarat. The communities living in Liguasan Marsh are generally impoverished and difficult to access due to remoteness and internal conflicts between rebel and government forces over several years.

### Diagnosis of yaws

The diagnosis of yaws was based on the combination of cutaneous and serologic signs. The clinical history of skin lesions resembling yaws, treatment, sexual contact, or high-risk behavior were also considered. Serologic tests can not distinguish between yaws and syphilis and definitive diagnosis would require the polymerase chain reaction (PCR) test. [11]

The study adopted the WHO Yaws case definitions for suspected, confirmed, imported, index case and contacts. [12] The case definitions of primary, active, secondary, latent, and tertiary yaws were based on the combination of cutaneous and serologic signs. The clinical history of skin lesions resembling yaws, treatment, sexual contact or high-risk behavior were also considered. If the non-treponemal and treponemal antibodies of the DPP test were both positive and yaws-like skin lesions were present, the patient was considered an *active yaws* case. If skin lesions were not present but both antibodies were detected, the patient was considered a *latent case*. If skin lesions were not present and only Treponemal antibodies were reactive (i.e. negative non-Treponemal antibody), the patient was considered a *past or treated yaws* case. *(S1 Table)* [13,14]

### Sample size

Based on the latest community skin survey from 1999–2000 in Maguindanao province and a serologic survey in Cotabato and Maguindanao in 2013 [8], we assumed the estimated prevalence of yaws to be 12%. The current study targeted 483 schoolchildren for each province, a total of 1449 children. This sample size was required to estimate the prevalence of yaws in this population within the interval 12 ± 3% with a 95% confidence level.

### Study approach

We followed the WHO recommendation for the detection and survey of yaws: In villages for which there is limited information on yaws, baseline clinical and serological surveys of children aged less than 15 years should be carried out to determine the burden. [12] We also aimed to detect latent and past yaws and therefore included household members of schoolchildren in our screening surveys as well as community members. Latent yaws, a condition that may persist especially among adults in an endemic area, can be a source of transmission to uninfected children when active yaws recurs. [15]

We also adopted a Community Skin Health approach in order to detect yaws. This approach had been used successfully to detect hidden cases of leprosy in other parts of the country by sensitizing the community leaders and the public to the importance of skin health, providing training of local health personnel, involving public schools, and by providing medications during free outreach skin clinics. [16]

### Social preparation phase

**Fig 1:**
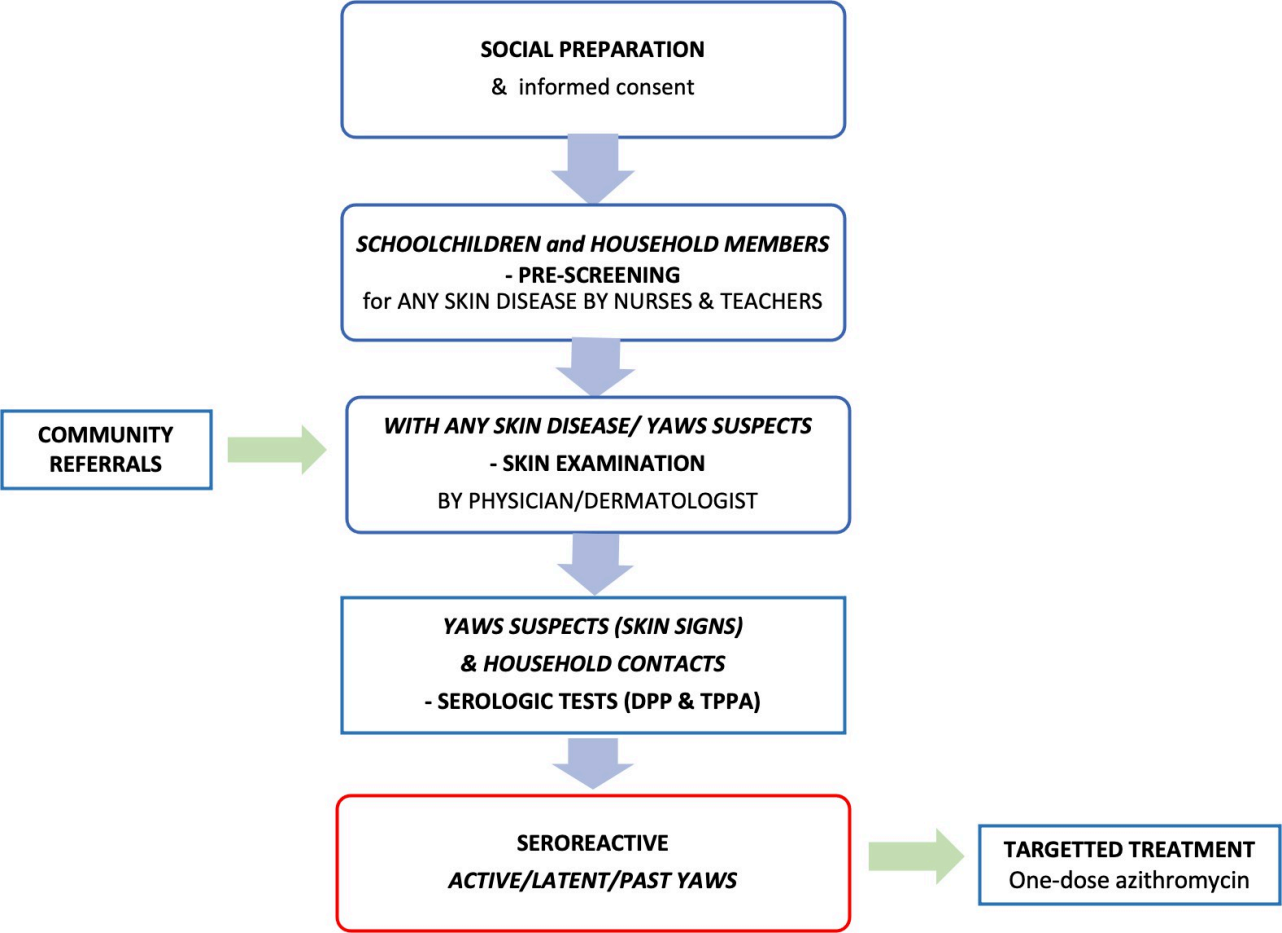
Study procedure flowchart.

Preceding the surveys, we conducted orientations and consultative meetings about the study and yaws for key stakeholders such as local leaders, local health personnel and school nurses in the municipalities involved. School nurses were trained by the investigator on how to inspect the children for skin diseases. Thereafter, the health personnel conducted orientations for teachers and parents in the selected schools. Parents and household members were invited by teachers and school nurses to the scheduled skin check-ups. Information materials on yaws and other common skin diseases were developed for the social preparation phase. The Yaws Recognition Booklet for Communities [17] was translated into the three languages spoken in the study sites (Filipino, Visayan, Hiligaynon) with permission from the WHO. Additional photographs of yaws among Filipino children were added to the booklets and flipcharts and made available to the study site leaders and health personnel, school administrators and teachers. PowerPoint presentations about yaws were also shown to the parents and children in schools. School teachers instructed the elementary school students to report household members who had any skin disease, guided by flyers with colored photographs of skin diseases including leprosy and yaws. Written informed consent from parents or guardians and written assent from children aged 6–18 years old were secured prior to the survey. (Fig 1)

### Pre-screening of students

School nurses pre-screened the students for any pathologic skin lesions especially for yaws. All those with any skin disease or with yaws-like skin lesions were instructed to have a skin check-up with the study physician on scheduled dates.

### Skin examinations and serological tests

During the scheduled free skin clinics, there were field teams composed of local health physicians and school nurses who conducted skin examinations of students, non-schooling children and adults who were household contacts, and community members. Village health workers and midwives brought community members who they suspected of having yaws or had significant skin diseases. The free skin consultations were held in designated rooms of the public schools or in the rural health centers.

Yaws suspects were identified based on skin signs such as yaws-like papules, small ulcers, pyodermatous lesions, multiple atrophic scars, and palmoplantar keratodermas. If an on-site dermatologist (BLD) was not present, store-and–forward teledermatology referrals were made by the field team for all cases of suspected yaws, leprosy, and pathologic skin diseases. Assigned integrated mobile phone cameras were used. WhatsApp or encrypted email were used to transmit images and send information. Other members of the study sites who wished to have skin check-ups were also included.

All those who were diagnosed by the physicians as yaws suspects were invited to undergo the serologic tests on-site. The field team also invited and examined all remaining household members as close contacts of yaws suspects because they were considered potential latent yaws cases. Two serological tests were performed on yaws suspects and their household contacts: a point-of-care test called the Dual Pathway Platform for Syphilis Screening and Detection (DPP–Chembio Company) and the *Treponema Pallidum* particle agglutination test (TPPA). Venous blood specimens drawn from the antecubital vein or fingerprick blood were used for the DPP tests. Whole venous blood specimens were collected for TPPA and transported to Cotabato Regional and Medical Center for processing and reading.

### Treatment

All confirmed active and latent yaws cases were treated with one dose of azithromycin. The pediatric dose of azithromycin single dose was 30 mg/kg body weight (maximum 2 grams) and the adult dose was a single dose of 2 grams. [18] Yaws cases were endorsed to the municipal health office or rural health physician for monitoring. All other significant skin diseases were managed either on site or at the local health center.

Contacts of yaws cases were also screened for skin lesions, underwent serological tests, and were given a single dose of azithromycin.

## Results

### Participants

A total of 2779 persons participated in the study: 2291 students, 393 household members, and 95 community members. The mean age was 11.7 years (SD 9.76). Among the participants, 2532 (91.1%) were children of 14 years old and younger. The majority were females (56%), 43.6% were males, and for 0.5% of participants gender was not specified. *(Tables 1 and S1)*

**Table 1:** Demographic characteristics of all participants.

	MAGUINDANAO		SULTAN KUDARAT		COTABATO			
**Gender**	Total	%	Total	%	Total	%	Total	%
Male	160	41.7	718	44.5	333	43.4	**1211**	**43.6**
Female	224	58.3	897	55.5	434	56.6	**1555**	**56**
No info	0	0	5	0.3	8	1	**13**	**0.5**
**Total**	**384**	**100**	**1615**	**100**	**767**	**100**	***2779***	***100***
**Age range (years)**								
0–4	14	3.6	13	0.8	24	3.1	51	1.8
5.- 9	92	24	839	51.8	356	45.9	1287	46.3
10. - 14	194	50.5	681	42	319	41.2	1194	43
15–19	12	3.1	23	1.4	9	1.2	44	1.6
20 & above	71	18.5	59	3.6	65	8.4	195	6.9
Unknown	1	0.3	5	0.3	2	0.3	8	0.3
**Total**	**384**	**100**	**1620**	**100**	**775**	**100**	***2779***	***100***

### Students

The total number of students screened for skin diseases was 2291 out of 6113 (37.5%) enrollees of Grade 1–6. The majority of the students were from Sultan Kudarat province (1387, 60.5%). Cotabato had 651 students (28.4%) and Maguindanao had 253 (7.5%). One elementary school in Sultan Kudarat had the highest number of student participants (n = 1090) comprising 55.4% of its enrollees. The mean age was 9.5 years (SD 2.2). Majority (98.5%) of the students were between 5 and 14 years old. Most of the students were females (54.7%) and 44.8% were males, while gender was not specified in 0.5%.

### Clinico-seroprevalence of yaws cases among students

A total of 2291 students in 9 schools participated in the survey. Among these students, 122 (5.3%) were considered yaws suspects based on the presence of skin lesions similar to yaws such as recurrent furuncles, abscesses, or multiple atrophic scars.

Ninety-five of the 122 yaws suspects (77.9%) consented to have blood tests. Only one student had confirmed active yaws. The 5-year-old girl from Site F Sultan Kudarat province had dry scaly papules on the dorsa of her feet and positive serologic tests for both Treponemal and non-Treponemal antibodies.

In the elementary school of Sultan Kudarat province where this confirmed case was detected, the prevalence was estimated at 1 yaws case per 1090 students screened (0.1%). Among all Sultan Kudarat schoolchildren (3 schools), yaws was estimated to be 1 / 1387 (0.1%) students screened.

The prevalence of active yaws among the gradeschool children surveyed in all study sites of Liguasan Marsh area (9 municipalities, 3 provinces) was 1 case among 2291 students screened (0.04%). *(Table 2)*

**Table 2:** Clinical & serological results of schoolchildren screened (N = 2291).

	**No.**	**%**
With any skin disease	665	29
Yaws suspects	122	5.3
Serological tests done	95	4.1
Active or Latent Yaws: Reactive Trep & non-Trep Abs	1	0.04
Past/Treated Yaws: Reactive Trep Ab, Negative non-Trep Ab	0	0
Negative for yaws: Negative Trep Ab, Reactive non-Trep Ab	2	0.1
Negative for yaws: Negative Trep Ab & non-Trep Abs	94	4.1
Active Yaws	1	0.04

Trep = Treponemal; Ab = Antibody

### Yaws cases detected among household members and community members

There were 393 household members of students and 95 community members screened for skin diseases. Only 39 household members and 16 community referrals with suspicious skin lesions consented to have blood tests done. Active, latent, and past yaws cases were detected among them. The details of the yaws cases are found in a separate article. [9]

**Fig 2:**
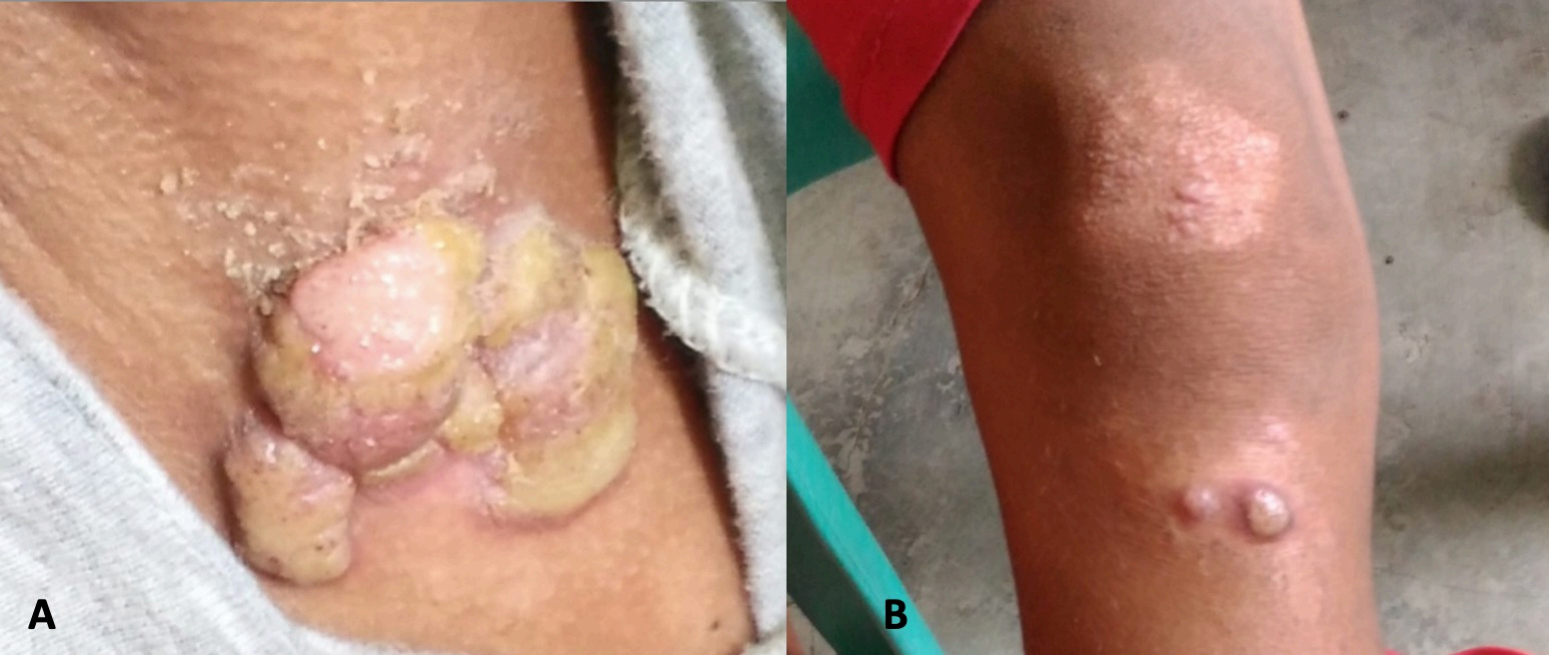
Index yaws case: 10 year old boy with large, “moist cauliflower” papillomas on left axilla (A), papulosquamous plaque and yellow-crusted nodules of secondary yaws on the leg (B) [9].

Three children from the community Site H, Cotabato had active yaws. (Fig 2) Eight latent cases were detected only among adults: 6 were household contacts of students from the selected schools and 2 were from the community. The two cases of past or treated yaws were also household contacts of students from the selected schools. These 2 adult household contacts from Maguindanao and Cotabato were only reactive to the Treponemal antibody in the DPP assay but with TPPA titers of 1:320. Since they had negative non-Treponemal antibody results, they were assessed to be past or treated yaws according to the WHO definition. Due to the high Treponemal antibody titers, however, it is also possible that they were latent cases of yaws. Both recalled having yaws-like skin lesions but no treatment in the past. One 42-year-old female had atrophic scars on the knees while the other 42-year-old female recalled a positive VDRL in the past but had no yaws-like skin lesions. The results of the DPP and TPPA were all concordant for Treponemal antibodies. Five participants were not reactive for Treponemal antibodies and were only positive for non-Treponemal antibodies. They were not considered yaws cases.

Over-all, among the children aged 14 years old and below, we detected active yaws in 4 out of 2532 children (0.2%) and no latent or past cases. Among the 2779 participants screened, latent yaws was found only in 8 adults (0.3%) while past yaws was detected in 2 adult household members (0.07%).

The summaries of survey findings are in Tables *3* and S3.

**Table 3:** Distribution of participants examined and yaws cases detected, by type of participant.

Type of Study Participant	Screened for skin disease	with Skin Disease		Yaws suspects & contacts		Serologically tested		Active yaws	Latent yaws	Past yaws	Total Cases
	N	No.	%	No.	%	No.	%				
Students	**2291**	665	29.0	122	5.3	95	4.1	1	0	0	**1**
Household members	**393**	250	63.6	61	15.5	39	9.9	0	6	2	**8**
Community Referrals	**95**	55	57.9	18	18.9	16	16.8	3	2	0	**5**
**Total**	**2779**	**970**	**34.9**	**201**	**7.2**	**150**	**5.4**	**4**	**8**	**2**	**14**

### Municipalities endemic for yaws

Two out of 9 municipalities (Site F, Sultan Kudarat and Site H, Cotabato) had confirmed active yaws cases. These two municipalities had no previous reports of yaws cases from the past two studies by Dofitas and Kalim. [8]

However, there were adults with a history of yaws and seroreactivity to *Treponema pallidum* and non-treponemal antibodies indicating possible latent yaws. These cases were found in 2 other municipalities in Maguindanao and Cotabato. (Site A, Maguindanao; Site I, Cotabato).

Datu Piang municipality in Maguindanao had numerous yaws cases reported by Dofitas in 1999 during a community skin survey. [19] However, no active yaws cases among children or adults were found in this municipality in the current survey.

**Fig 3:**
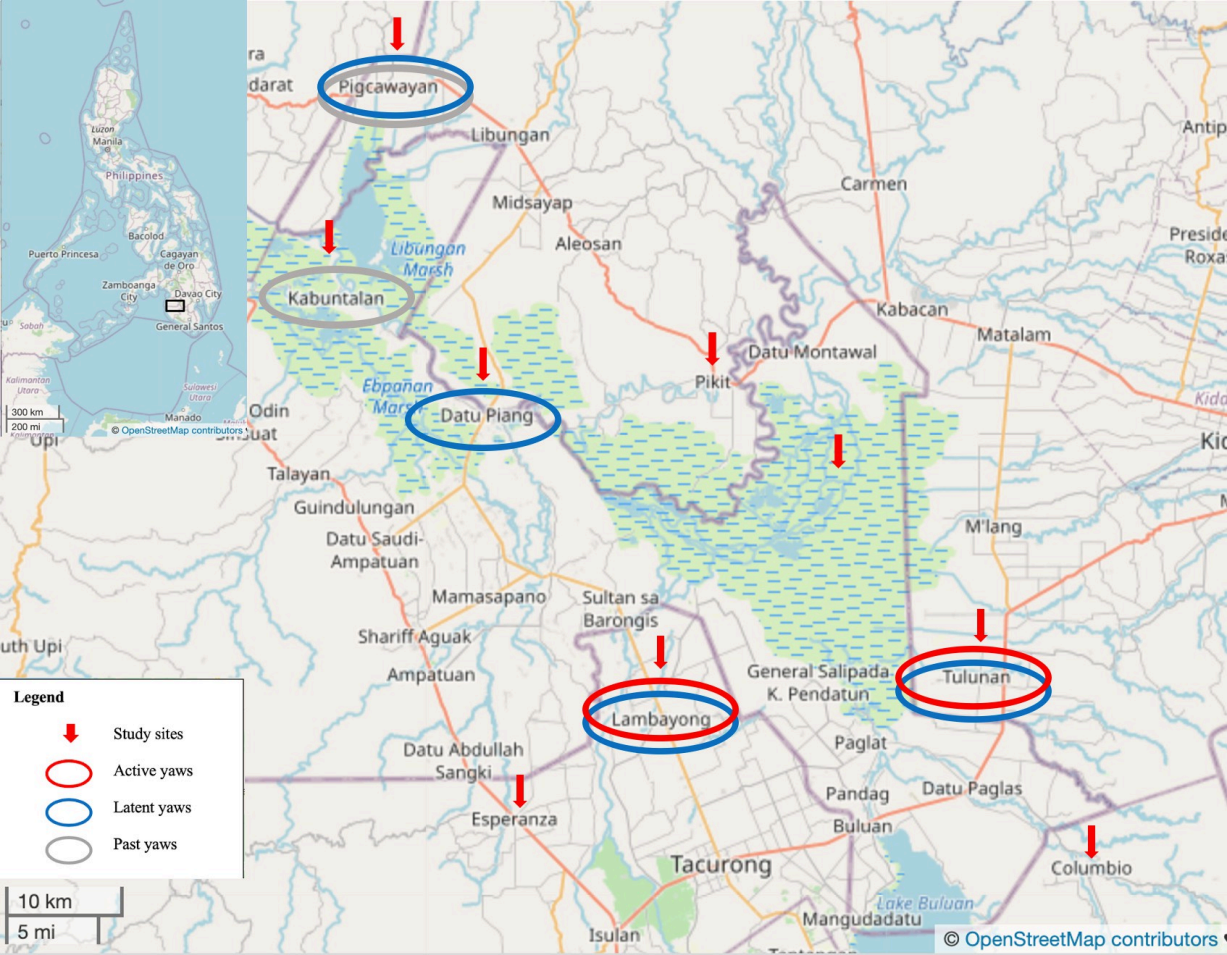
Location map of municipality study sites and yaws cases detected in Mindanao Island, Philippines (Contains information from OpenStreetMap and OpenStreetMap Foundation, which is made available under the Open Database License) [20].

Overall, five out of 9 study municipalities located in all three study provinces at the Liguasan Marsh area can be considered endemic for yaws. (Fig 3)

According to key informants (e.g. local healthcare workers and residents), other municipalities in the Liguasan Marsh area and BARMM were reported to have yaws. Doctors who were already working in the study provinces reported to the principal investigator (BLD) that they had seen possible yaws cases in the past ex. Cotabato province: Pikit, Pigkawayan, Midsayap; Maguindanao, Sultan Kudarat province, and other municipalities in Liguasan Marsh and Lake Buluan. Local efforts to treat yaws cases with one-dose benzathine penicillin injections were actually done by a municipal health officer in the 80’s and the 90’s in Maguindanao. Another municipal health officer from Pikit, Cotabato informed this investigator that a private doctor had gone around injecting yaws cases there some years ago.

## Discussion

In this paper, we report the first exploratory prevalence study of yaws in selected communities in the Philippines since the evaluation of the yaws eradication program in 1963. [21] We confirmed the presence of active, latent, and past yaws through clinical and serologic methods recommended by the WHO yaws programme. The prevalence of active yaws among schoolchildren was low at 1 case per 2291 (0.04%). An additional 3 active yaws cases were detected among children in the community, making a total number of 4 active yaws cases (0.2%) among the 2532 children aged 14 years old and below who participated. The estimated seroreactivity among 95 children aged 0–14 years old was 4.2%. Five of the 9 municipalities studied were found to be endemic for yaws. Latent yaws was found in 0.3% of participants.

Five patients among the 150 tested (3.3%) were reactive only to non-treponemal antibodies. They could be early yaws infection cases that had not developed Treponemal antibodies yet or they may have had other disease conditions such as leprosy, tuberculosis, or autoimmune diseases.

The WHO criteria states that one confirmed case of yaws makes the community endemic. [18] Such is the criterion because yaws is earmarked for eradication and even one active case can easily infect contacts in the community. This study showed that yaws was not eradicated in the Liguasan Marsh area and has been in existence in these communities for as long as the adult residents can remember. This study provided the basis for adding the Philippines to the list of countries that are endemic for yaws, although yaws was limited to the study sites in Mindanao. The major finding of this study is the confirmation of active and latent yaws in 5 out of the 9 municipalities in provinces where Liguasan Marsh is found.

### Lessons for countries previously endemic for yaws

Countries previously endemic for yaws can learn from the case detection and survey methods used in our study. The search for yaws is challenging when its presence and appearance is unknown to the health sector and the public. To detect yaws during this study, the investigators had to rely on the local residents’ knowledge of yaws (called "*bakataw"* among Maguindanaoans), and orient the health workers and schools about yaws using photographs from the Yaws Recognition Booklet for Communities, and file photographs of the investigator (BLD) from previously encountered yaws cases in Liguasan Marsh. This study adopted a strategy called Partners in Leprosy Action (PILA) which was developed and successfully used by the Philippine Leprosy Mission. [16] PILA utilized community skin health as an entry point for detecting yaws cases. It was the most strategic approach because it did not single out yaws as the problem, thus reducing the apprehension of affected individuals to consult for any skin problem. This strategy was also able to detect and treat other diseases of public health importance such as leprosy, cutaneous larva migrans, and other pathologic skin conditions needing attention.

We followed the actions recommended by the WHO for countries with a previous history of yaws in the 1950s but no report since 2013 (current status unknown). The first step would be to assess the status through enhanced public awareness and to review past and existing records. Clinical surveys among children aged under 15 years should then be conducted in previously endemic areas and those with suspected lesions should be tested serologically. [22]

We had no surveillance data on yaws since the 1970s and only had historical records and yaws sightings to guide our study site selection. Non-probability sampling was used in selection of the municipalities to increase the chances of detecting yaws. Investigating areas with the highest risk for yaws (i.e. remote villages, yaws sightings) would make more efficient use of our limited research resources. More extensive prevalence studies will be justified if yaws cases are detected during these exploratory surveys.

We also interviewed long-time residents and health workers about yaws sightings. “Rumor” reports and rumor registries have been successfully used for yaws campaigns in other countries such as Africa and India. [22] Long-time residents and village health workers of our study sites recalled yaws and children with yaws-like lesions in their villages as well as other parts of their provinces.

In Cameroon, Boock noted that passive case detection in local clinics had a low number of yaws cases detected while community-based case detection had higher yield. The investigators found out that the most successful means of detecting yaws were “mass outreach programs designed to educate the public about neglected tropical diseases found in the region and follow up school-based screening programs. These programs were supported by local chiefs and traditional healers and found to be the best way of increasing community awareness about yaws, motivating community health workers to participate in outreach, and fostering trust in the free medical treatment being provided.” The outreach educational programs were described as well-planned and culturally sensitive. [23]

### Awareness of active and latent yaws

The clinical appearance of the active yaws cases detected during this study had more of the "moist cauliflower" appearance compared to dry, yellow-crusted nodules and skin ulcers of African children in the Yaws Recognition Booklet. All four active yaws cases detected had the whitish, dry, papulosquamous or "pebbly" appearance of yaws and scars of healed yaws lesions. The moist, yellow-crusted, and dry papulosquamous types of yaws were simultaneously found in two of our yaws cases. The moist type of yaws skin lesions may be a result of the wet and humid environment of the Liguasan Marsh area. The health workers and clinicians should be aware of these variety of yaws clinical morphologies among Filipinos so that yaws can be suspected and confirmed serologically.

Although only two municipalities had confirmed active secondary yaws cases (i.e. 4 pediatric cases in Lambayong, Sultan Kudarat province and Tulunan, Cotabato), four municipalities had eight latent yaws cases among adult household contacts and community members. There is a possibility of latent syphilis infection among adults and older adolescents and PCR would be required to distinguish between yaws and syphilis. The diagnosis of latent yaws is more likely due to the historical and current presence of yaws in the area, [8] the low prevalence of syphilis, [24] and the low-risk profile for sexually transmitted infections of these affected individuals. The non-infectious latent phase may last for a lifetime but recurrences of active yaws lesions may occur and transmit the infection. Undetected and untreated latent cases have been the source of continued endemicity after mass drug administration during the yaws eradication campaign in the 1950s. [25]

### Prevalence of yaws

According to the Yaws Fact Sheet of WHO [1], “In 2013, there were 13 countries known to be endemic with yaws. Since then, through intense surveillance activities, two additional countries reported confirmed cases (Liberia and Philippines) and three countries reported suspected yaws cases (Colombia, Ecuador and Haiti. In the South-East Asia Region of WHO, Indonesia and Timor-Leste remain endemic. Since 2004, India has reported no new cases. In the Western Pacific Region, three countries remain endemic–Papua New Guinea, the Solomon Islands and Vanuatu—with the addition of the Philippines in 2017." In 2021, one case of yaws in a 5-year old child was reported in Malaysia, a previously endemic country in the 1950s. [26]

The over-all prevalence of active yaws in the Liguasan Marsh study sites was low compared to other yaws-endemic countries although this is probably due to our study’s limited sampling. The distribution of yaws is known to be in pockets of a geographical area, usually in remote and inaccessible villages. Therefore, it is possible that the randomly selected schools in our survey were in low-endemic sites and that high-endemic sites were missed due to the limited sampling. Our findings are much lower than community skin survey findings of Dofitas in Datu Piang, Maguindanao in year 1999 (11.7% with signs of active yaws and scars) and the serologic findings of Kalim in year 2013 (13% among women and children). [8] Overestimation of yaws prevalence is likely when reports are based on clinical signs alone.

The current rates of yaws may be low, but it is still a public health concern considering that yaws is a highly infectious and chronic skin disease that can potentially infect several more individuals by skin-to-skin contact or minor breaks in the skin. All of the active yaws cases detected were already in school and potentially infecting other children and adult contacts. There were 12 active and latent yaws combined in the study areas that reflect the infectious cases and potential sources of transmission.

Several active yaws cases were detected in the 1999–2000 skin survey by Dofitas. However, there have been no government-initiated public health control measures taken. There were efforts by a municipal health officer and private doctor to treat yaws cases in the past. These interventions may have reduced the number of yaws cases in those communities over the years. However, there was no systematic public health control or eradication program in place that could educate the public and health personnel about yaws and its consequences, how it could be prevented and treated. The WHO’s inclusion of yaws as a Neglected Tropical Disease that is targeted for eradication has given more government awareness and the impetus for such a control and eradication program to be established in the Philippines.

The factors that contribute to the continued presence of yaws in the Philippines may be the following:

1. The Philippines was highly endemic for yaws from the northern to southern regions prior to the eradication campaign of the 1950s. After yaws was almost eradicated at the end of the campaign, yaws was not given due attention by the local public health system, enabling the remaining yaws cases to spread the infection.
2. Owing to the proximity and close relationship between endemic Indonesia and the Southern Philippines, the likelihood of yaws cases entering our country is quite high.
3. Liguasan Marsh area has accessibility and security problems because armed groups reside in this area and skirmishes with government troops disrupt basic services, especially health, and perpetuate poverty. Poverty and lack of information among the communities residing there have most likely contributed to the resurgence and spread of yaws. The marshy environment also promotes the growth and transmission of microorganisms among the dwellers there.

Key informant interviews of healthcare workers and residents revealed that yaws had been sighted in other parts of the provinces included in the survey. With the mobility of the Filipino population, yaws may easily spread to other parts of the country.

Yaws may be present in other parts of the Philippines but may have been unreported because this disease is no longer recognized by health professionals and health workers. Yaws was also thought to have been eradicated in our country and was therefore forgotten as a public health concern and even as a reportable disease. This is apparent by the lack of records of yaws at the Department of Health beyond 1973 or any publication about yaws in the Philippines since the evaluation report of the yaws eradication program was published in 1965.

### Limitations

The prevalence of active yaws among schoolchildren in our study is lower than those previously reported in other countries, however, the scope of our study was also limited in sample size (i.e. one school per municipality) which may have caused an underestimation of the prevalence. Community surveys especially in remote villages within the marsh could likely yield higher case detection rates.

The timing of school surveys was also a limiting factor. The school-based surveys were only possible within January to March 2017 due to logistical delays and conflicts with other school activities in November, December 2016, and April 2017. For school surveys to be successfully implemented, the preparatory activities should be immediately followed by the implementation of the survey. School teachers and school nurses were the main field team personnel. Their training for the study plus the orientations of teachers, parents, and children must be scheduled close to the implementation dates. The best time to initiate a school-based survey in the Philippines would have been early in the schoolyear such as June or July so that there would be more time before December (Christmas season) to collect data.

Accessibility issues and peace and order problems of the more remote and impoverished villages of Liguasan Marsh did not allow for the search for yaws cases in the areas where they were most likely to exist: “where the road ends”.

## Conclusion

This study confirmed that the Philippines is endemic for yaws but at a low level in the schools and communities surveyed. The lack of proper disease surveillance after the eradication campaign in the 1960’s has led to the resurgence of yaws. Yaws could be unreported in other previously endemic countries as well because the skin and bone signs are not recognized anymore.

At present, the Department of Health is continuing the search for any case of active yaws in the remaining regions of the country through a follow-up study on active yaws surveillance and clinico-seroprevalence surveys of remote, previously endemic villages and among indigenous peoples (IP). New yaws cases have been detected in the Liguasan Marsh and, recently, this study’s principal investigator (BLD) found yaws in one IP village of the northern region of the Philippines. Active surveillance is needed to determine the extent of yaws in the Philippines and to help develop a strategy to eradicate yaws by 2030. We should increase our diagnostic capacity to distinguish between yaws and syphilis. Once more epidemiological surveys are completed, the yaws control and eradication program can be formulated as either a localized or nationwide program.

## Supporting information

S1 Table Case definitions of yaws. (DOCX)

S2 Table Distribution of age and sex of students, household members, and community referrals. (DOCX)

S3 Table Summary of clinical & serologic results of all participants. (DOCX)
