# Supplementary material for: Yaws in the Philippines: A clinico-seroprevalence study of selected communities in Mindanao
Source: PLoS Negl Trop Dis. 2022 Jun 1;16(6):e0010447. doi: 10.1371/journal.pntd.0010447 (PMC9159601; doi:10.1371/journal.pntd.0010447)
Supplement: S1 Table — (DOCX) [file pntd.0010447.s001.docx]

**S1 Table.** **Case definitions of yaws (all criteria must be fulfilled)**

| **Classification of Yaws** | **Clinical features** | **Clinical history** | **Serologic tests** |
| --- | --- | --- | --- |
| Active  (Primary or secondary) | Single or multiple reddish, small papule/s, verrucous papule/s or nodules, reaching up to 5 cm in diameter, often ulcerated, resembling a raspberry or pickled cauliflower, and covered with yellow crust  circinate, macular,maculo-squamous, papular/ nodular resembling syphilids | No history or evidence of syphilis, previous sexual contact, or maternal-child transmission of syphilis | Reactive treponemal  AND  non-treponemal antibody tests (ex. DPP T1 & T2/ TPPA/ TPHA and VDRL/RPR) |
| Inactive (Tertiary) | Cutaneous manifestations- enlarging, painless nodule with necrosis, ulceration;  -Ulcer has well-defined edges, indurated base, granulation, yellowish slough  -Palmoplantar hyperkeratosis  Extra-cutaneous: Skeletal (gummatous/ non-gummatous), Juxta-articular nodules, Saber tibia, boomerang legs, gangosa | No history or evidence of syphilis, previous sexual contact, or maternal-child transmission of syphilis | Reactive confirmatory test for *T pallidum* (DPP T1/ TPPA/ TPHA)  but  Negative non-treponemal antibody test |
| Latent | NO visible signs of active yaws; positive or negative history of skin lesions compatible with yaws; +/- scars | No history or evidence of syphilis, previous sexual contact, or maternal-child transmission of syphilis | Reactive treponemal and non-treponemal antibody tests (ex. DPP T1* & T2**/ TPPA***/ TPHA****) |
| Past or treated treponemal infection (either syphilis, yaws, pinta) | NO visible signs of active yaws; positive or negative history of skin lesions compatible with yaws, syphilis, or pinta | +/- history or evidence of syphilis, yaws, or pinta, previous sexual contact, or maternal-child transmission of syphilis | Reactive confirmatory test for *T pallidum* (DPP T1/ TPPA/ TPHA)  but  Negative non-treponemal antibody test |
| False positive for yaws | Visible signs of active yaws or scars OR with a history of yaws-like lesions | No history or evidence of syphilis, previous sexual contact, or maternal-child transmission of syphilis | Reactive non-treponemal test (DPP T2/ VDRL/RPR)  but Negative confirmatory treponemal antibody test (DPP T1 or TPPA, TPHA) |

*DPP T1= Dual Pathway Platform Syphilis Screen and Confirm Assay -Recombinant Treponemal antigen

**DPP T2 = Dual Pathway Platform Syphilis Screen and Confirm Assay -Synthetic Non-treponemal antigen

***TPPA = *Treponema pallidum* Particle Agglutination test

****TPHA = *Treponema pallidum* Hemagglutination test
