# Supplementary material for: Yaws in the Philippines: A clinico-seroprevalence study of selected communities in Mindanao
Source: PLoS Negl Trop Dis. 2022 Jun 1;16(6):e0010447. doi: 10.1371/journal.pntd.0010447 (PMC9159601; doi:10.1371/journal.pntd.0010447)
Supplement: S2 Table — (DOCX) [file pntd.0010447.s002.docx]

**S2 Table. Distribution of age and sex of students, household members, and community referrals**

|  | **STUDENTS** | | **HOUSEHOLD MEMBERS** | | **COMMUNITY REFERRALS** | | **TOTAL** | |
| --- | --- | --- | --- | --- | --- | --- | --- | --- |
| **n** | **2291** |  | **393** |  | **95** |  | **2779** |  |
| **Age range (years)** | **No.** | **%** | **No.** | **%** | **No.** | **%** | **No.** | **%** |
| 0-4 | 0 | 0.0 | 47 | 12.0 | 4 | 4.2 | 51 | 1.8 |
| 5-9 | 1,162 | 50.7 | 110 | 28.0 | 15 | 15.8 | 1287 | 46.3 |
| 10-14 | 1,094 | 47.8 | 84 | 21.4 | 16 | 16.8 | 1194 | 43.0 |
| 15-19 | 27 | 1.2 | 14 | 3.6 | 3 | 3.2 | 44 | 1.6 |
| 20 & above | 0 | 0.0 | 138 | 35.1 | 57 | 60.0 | 195 | 7.0 |
| No info | 8 | 0.3 | 0 | 0.0 | 0 | 0.0 | 8 | 0.3 |
| **Sex** |  |  |  |  |  |  |  |  |
| Male | 1026 | 44.8 | 151 | 38.4 | 34 | 35.8 | 1211 | 43.6 |
| Female | 1253 | 54.7 | 242 | 61.6 | 60 | 63.2 | 1555 | 56.0 |
| No Info | 12 | 0.5 | 0 | 0.0 | 1 | 1.1 | 13 | 0.5 |
