# Supplementary material for: Yaws in the Philippines: A clinico-seroprevalence study of selected communities in Mindanao
Source: PLoS Negl Trop Dis. 2022 Jun 1;16(6):e0010447. doi: 10.1371/journal.pntd.0010447 (PMC9159601; doi:10.1371/journal.pntd.0010447)
Supplement: S3 Table — (DOCX) [file pntd.0010447.s003.docx]

**S3 Table. Summary of clinical & serologic results of all participants**

| **Type of Study Participant** | **No.** | **% screened (N=2779)** | **% with Skin Disease (N=970)** | **% yaws suspects & contacts (N=201)** | **% serologically tested (N=150)** |
| --- | --- | --- | --- | --- | --- |
| Invited | 6113 |  |  |  |  |
| Participated | 2779 |  |  |  |  |
| With skin disease | 970 | 34.9% |  |  |  |
| Yaws suspects & contacts | 201 | 7.2% | 20.7% |  |  |
| Confirmed yaws | 4 | 0.1% | 0.4% | 2.0% |  |
|  |  |  |  |  |  |
| Serologic tests done | 150 | 5.4% | 15.5% | 74.6% |  |
| Active or Latent Yaws: Reactive Trep & non-Trep Abs | 12 | 0.4% | 1.2% | 6.0% | 8.0% |
| Past/Treated Yaws: Reactive Trep Ab, Negative non-Trep Ab | 2 | 0.07% | 0.2% | 1.0% | 1.3% |
| Negative for yaws: Negative Trep Ab, Reactive non-Trep Ab | 5 | 0.2% | 0.5% | 2.5% | 3.3% |
| Negative for yaws: Negative Trep & non-Trep Ab | 131 | 4.7% | 13.5% | 65.2% | 87.3% |
|  | |  |  |  |  |

Trep = Treponemal; Ab = Antibody
